# Autofluorescence Is a Common Trait in Different Oceanic Fungi

**DOI:** 10.3390/jof7090709

**Published:** 2021-08-29

**Authors:** Eva Breyer, Markus Böhm, Magdalena Reitbauer, Chie Amano, Marilena Heitger, Federico Baltar

**Affiliations:** Department of Functional and Evolutionary Ecology, University of Vienna, 1030 Vienna, Austria; a00255710@unet.univie.ac.at (M.B.); a01441286@unet.univie.ac.at (M.R.); chie.amano@univie.ac.at (C.A.); a01540889@unet.univie.ac.at (M.H.)

**Keywords:** marine fungi, fluorescence in situ hybridisation, mycoplankton, fungal cultures, pelagic, fluorescence

## Abstract

Natural autofluorescence is a widespread phenomenon observed in different types of tissues and organisms. Depending on the origin of the autofluorescence, its intensity can provide insights on the physiological state of an organism. Fungal autofluorescence has been reported in terrestrial and human-derived fungal samples. Yet, despite the recently reported ubiquitous presence and importance of marine fungi in the ocean, the autofluorescence of pelagic fungi has never been examined. Here, we investigated the existence and intensity of autofluorescence in five different pelagic fungal isolates. Preliminary experiments of fungal autofluorescence at different growth stages and nutrient conditions were conducted, reflecting contrasting physiological states of the fungi. In addition, we analysed the effect of natural autofluorescence on co-staining with DAPI. We found that all the marine pelagic fungi that were studied exhibited autofluorescence. The intensity of fungal autofluorescence changed depending on the species and the excitation wavelength used. Furthermore, fungal autofluorescence varied depending on the growth stage and on the concentration of available nutrients. Collectively, our results indicate that marine fungi can be auto-fluorescent, although its intensity depends on the species and growth condition. Hence, oceanic fungal autofluorescence should be considered in future studies when fungal samples are stained with fluorescent probes (i.e., fluorescence in situ hybridization) since this could lead to misinterpretation of results.

## 1. Introduction

Natural autofluorescence is a process in which endogenous cell compounds i.e., aromatic amino acids, become fluorescent when excited with light in a specific wavelength. Depending on the type and origin of the fluorescent compound, the intensity of the emitted light can change with the morphological or physiological state of the observed cells or tissue and, hence, the organism [1].

Autofluorescence in fungi was first observed when human tissue sections, used to diagnose mycotic infectious diseases, were exposed to UV-light. Diverse fungi including Candida, Aspergillus, Blastomyces, Cryptococcus and Coccidioides, emitted fluorescent light, allowing their easy detection in infected tissue samples without prior staining [2,3]. Moreover, experiments with terrestrial-derived fungal cultures investigating autofluorescence in spores revealed differences in fluorescence intensities between fungal species, which might be related to different degrees of cellular viability [4]. In contrast, it was found that arbuscular mycorrhizal fungal structures were auto-fluorescent under blue and green light excitation despite their viability [5]. Likewise, fungal autofluorescence persisted after cell death following sample fixation, further demonstrating its diagnostic value for direct analyses of histological samples [6].

The origin of fungal autofluorescence is still not clear. It has been hypothesized that chitin could be the cause for fungal autofluorescence due to the similarities observed when fungal cells were stained with Calcofluor White (that binds to chitin in fungal cell walls) [5]. Recently, ergosterol, a membrane lipid found in fungal cell walls in the sub-kingdom of Dikarya used to quantify fungal biomass was suggested as another source of autofluorescence [7].

Previous research only focused on terrestrial and human-associated fungi. Recently, the important contribution of marine fungi to oceanic microbial food webs [8,9,10], biomass [11,12] and functional diversity [13,14] has been highlighted. Despite the ubiquitous presence of pelagic fungi in the oceanic water column, there are no investigations studying the existence of autofluorescence of oceanic fungi, and on the factors affecting the presence and intensity of this autofluorescence. To fill this gap of knowledge, we studied the autofluorescence of five marine pelagic fungi and investigated their autofluorescence at different growth stages. Furthermore, we also investigated the effect of varying nutrient concentrations and DAPI co-staining (a common DNA-stain in microbiology) on this fungal autofluorescence. Based on previous research, we hypothesized that marine fungi would be auto-fluorescent, similarly to certain terrestrial and human-associated fungi [2,3,5]. We also hypothesized that the autofluorescence intensity changes with species and physiological state, since it is possible that the production and distribution of auto-fluorescent compounds changes in response to growth and physiological state [1].

## 2. Materials and Methods

### 2.1. Cultivation of Marine Fungal Cultures

Four marine fungal cultures (*Metschnikowia australis*, *Rhodotorula sphaerocarpa*, *Sakaguchia dacryoidea, Blastobotrys parvus*) obtained from the Austrian Centre of Biological Resources (ACBR), and one fungal species (*Rhodotorula* sp.) isolated during the ‘Poseidon’ research cruise in 2019, were used to study the existence and intensity of autofluorescence in growth experiments (Table 1). All the five fungal species used (four yeasts and one hyphae-morphotype) were originally isolated from open ocean waters. The fungi were grown in the dark at room temperature on solid agar media containing (g/L): 10 g glucose, 5 g peptone, 3 g yeast extract, 3 g malt extract, 35 g artificial sea salts, 20 g agar and 0.5 g chloramphenicol.

**Figure 1:**
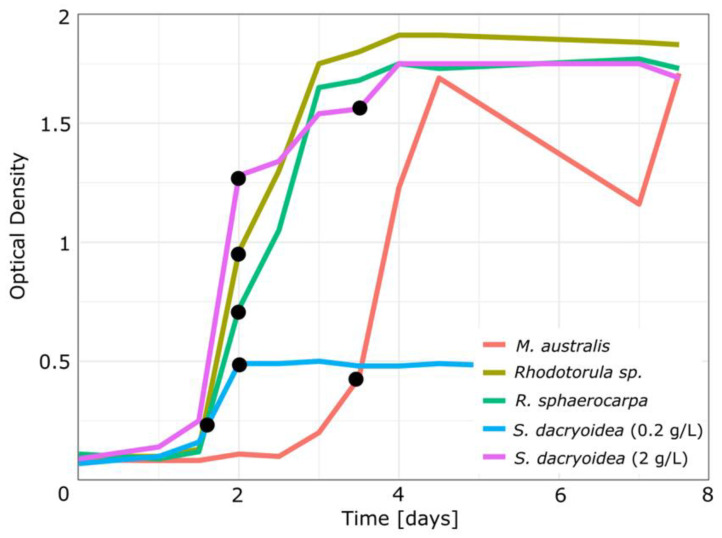
Growth of yeast cultures as indicated by optical density. Note: *S. dacryoidea* was cultured in two different nutrient concentrations (0.2 and 2 g/L glucose, blue and purple line, respectively). The other fungal species were grown with 2 g/L glucose. Black dots indicate the sampling times.

Aliquot samples of the yeasts were transferred and grown in liquid media (Table 2) to obtain samples at specific growth stages. All yeasts were cultured in the oligotrophic medium, and *S. dacryoidea* was additionally cultured in the eutrophic medium. The liquid cultures were incubated on a shaker incubator (Argo Lab, Ski 4, 140 rpm, Carpi, Italy) at room temperature under normal day-night light regime. The exponential growth stage was sampled in all species according to the daily measured optical density (UV-1800 Shimadzu spectrophotometer, λ = 660 nm, Kyoto, Japan). Additionally, *S. dacryoidea* was sampled in the stationary growth stage. Sampling of the different growth stages were based on previous experiments and on the shape of the growth curve (Table 3, Figure 1).

### 2.2. Sample Preparation to Investigate Fungal Autofluorescence

In the corresponding growth stages, 20 mL of the yeast cultures were sampled and fixed with 2% final conc. of formaldehyde (Sigma-Aldrich, 37%, St. Louis, MO, USA). Microscopic samples were prepared by filtering 250 µL of fixed culture, diluted in 5 mL MilliQ-water on GTTP filters (0.22 µm, 25 mm diameter, Merck Millipore, Burlington, MA, USA). Subsequently, filters were dried for 30 min and mounted with Vectashield (Vector Laboratories, H-1000, Burlingame, CA, USA) on a microscopic slide.

To further investigate the autofluorescence in hyphae-morphotype fungi, *Blastobotrys parvus* was diluted in artificial seawater (35 g/L sea salts), then filtered and mounted with Vectashield as described before.

To investigate fungal autofluorescence, samples were examined with a Zeiss Axio Imager 2 microscope (1250× magnification, Carl Zeiss, Jena, Germany) using four different channels and filter sets provided by Zeiss: DAPI (4′,6-diamidin-2-phenylindol, filter set 49); FITC (fluorescein isothiocyanate, filter set 44); DsRed (red fluorescent protein, filter set 43 HE); rhodamine (filter set 20 HE). For comparing potential species-specific differences in autofluorescence, each filter channel was analysed with fixed exposure times (2.6 s for DAPI, 5.2 s for FITC, 9.06 s for DsRed, 1.1 s for rhodamine) to obtain optimal results based on previous autofluorescence investigations. Here, we intentionally chose relatively long exposure times to examine autofluorescence. For usual fluorescence measurements, we used exposure times automatically calculated by the software (Axio Vision SE64-Re4.9, Carl Zeiss) of around 100–500 ms for Calcofluor-White (Sigma-Aldrich, St. Louis, MO, USA) and 150–500 ms for DAPI staining. Pictures were taken with an AxioCam MRm camera (Carl Zeiss).

## 3. Results and Discussion

*Sakaguchia dacryoidea* was grown in two different nutrient concentrations (0.2 and 2 g/L glucose) and sampled in the exponential and stationary growth stage (Figure 1). In both nutrient concentrations, *S. dacryoidea* entered the exponential growth phase after 1.5 d. In the media with less nutrients, the stationary phase was reached after 2 d, with a maximum OD of about 0.5. Conversely, at high nutrient concentrations, the *S. dacryoidea* yield was higher, reaching OD values in the stationary phase of about 1.8. It is noteworthy that all fungal species grown with 2 g glucose/L reached similar maximum biomass in the stationary phase.

**Figure 2:**
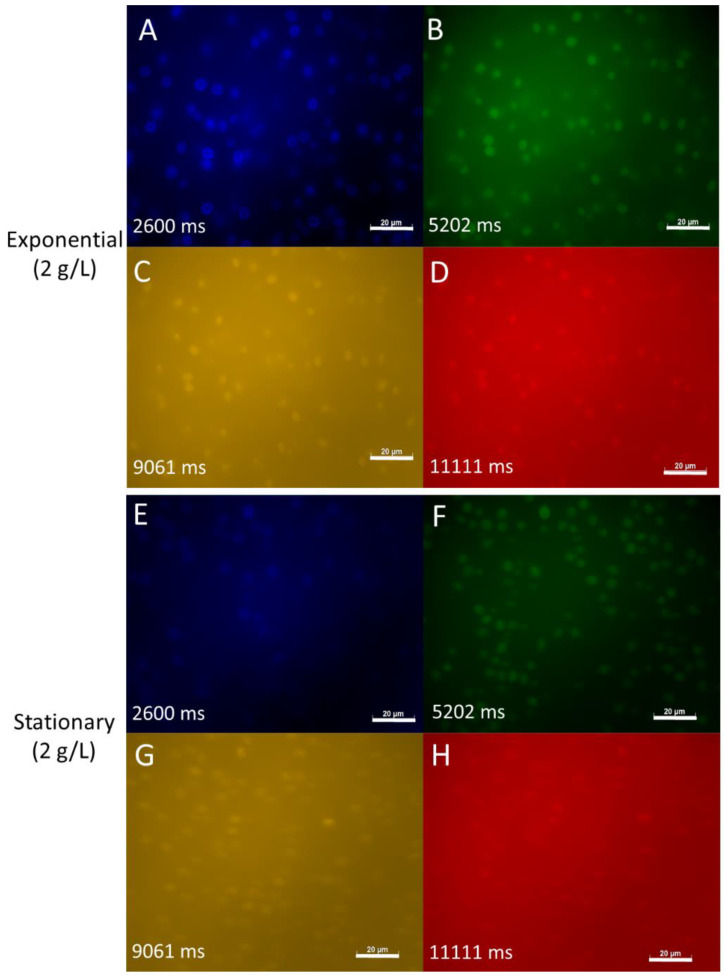
Autofluorescence of *S. dacryoidea* grown in the eutrophic medium and sampled in the exponential and stationary phase. (**A**,**E**): DAPI channel; (**B**,**F**): FITC channel; (**C**,**G**): DsRed channel; (**D**,**H**): rhodamine channel. Picture exposure times are depicted in milliseconds (ms). Scale bar = 20 µm.

When observed under the microscope, *S. dacryoidea* sampled in the exponential phase exhibited autofluorescence in all of the channels investigated (i.e., DAPI, FITC, DsRed and rhodamine). The strongest autofluorescence was detected in the DAPI channel as indicated by the shortest exposure time (in this case of 2.6 s) (Figure 2). The autofluorescence of *S. dacryoidea* was weaker in the FITC, DsRed and Rhodamine than in the DAPI channel.

To test whether the autofluorescence was affected by the growth stage we also examined it in the stationary phase of the same fungal culture (Figure 2). We found that the autofluorescence of *S.dacryoidea* became weaker in all observed channels compared to the exponential phase, indicating that nutrient limitation in the stationary phase affects marine fungal autofluorescence (Figure 2).

**Figure 3:**
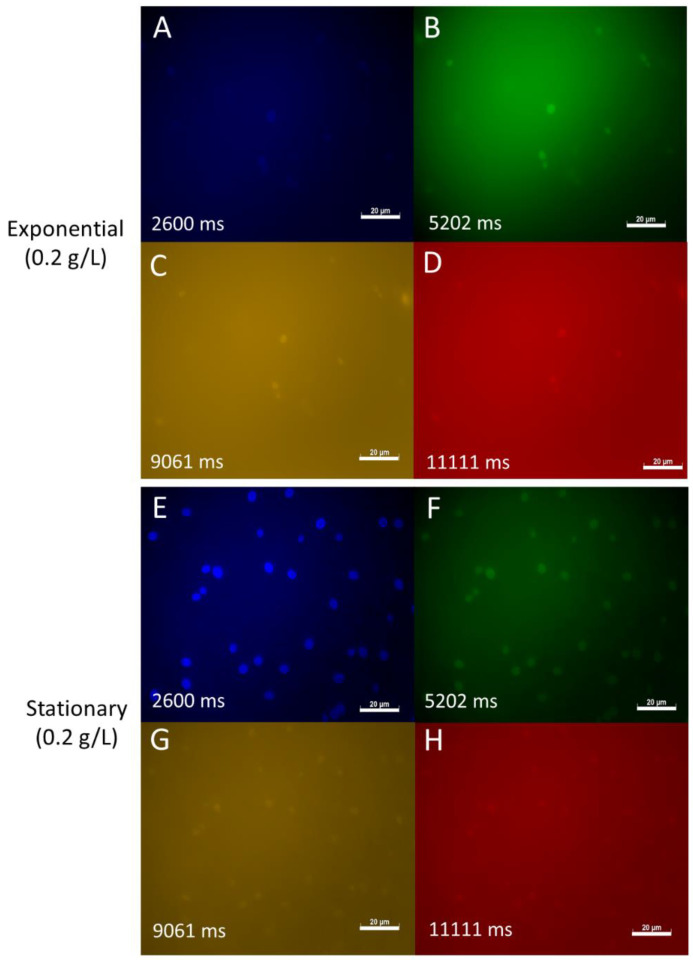
Autofluorescence of *S. dacryoidea* grown in the oligotrophic medium and sampled in the exponential and stationary phase. (**A**,**E**): DAPI channel; (**B**,**F**): FITC channel; (**C**,**G**): DsRed channel; (**D**,**H**): rhodamine channel. Picture exposure times are depicted in milliseconds (ms). Scale bar = 20 µm.

To gain more insight into the potential effect of nutrient concentrations on fungal physiology and autofluorescence, the autofluorescence of *S. dacryoidea* was analysed after culturing in a medium with reduced (10 times less) glucose concentration (i.e., 0.2 g/L) (Figure 3). In all of the analysed channels, the autofluorescence of *S. dacryoidea* in the exponential phase was slightly lower under lower nutrient conditions than at high nutrient concentrations (Figure 2 and Figure 3). However, in the stationary phase, the autofluorescence of *S. dacryoidea* increased again under low nutrient conditions (Figure 3).

**Figure 4:**
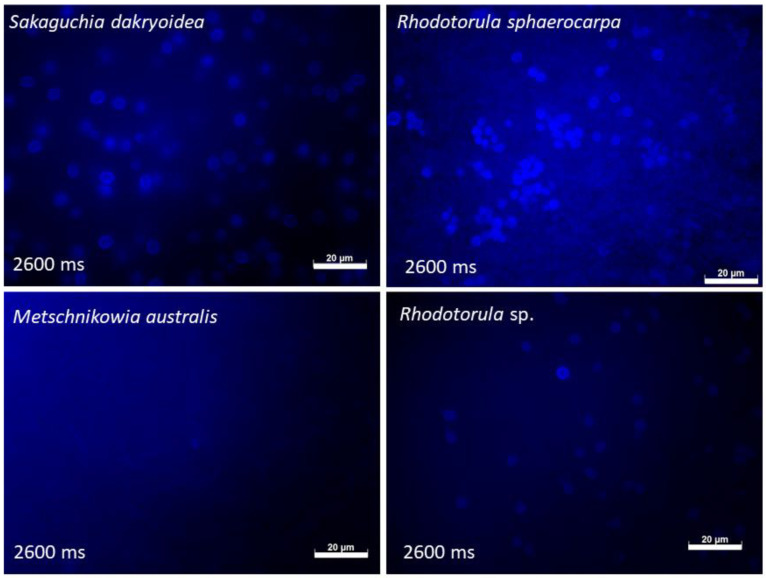
Autofluorescence of four marine yeasts grown in eutrophic medium and sampled in the exponential phase. All pictures were made in the DAPI channel, exposure times are depicted in milliseconds (ms). Scale bar = 20 µm.

To determine whether autofluorescence is a general phenomenon in oceanic fungi or whether it is species-dependent, we compared the autofluorescence of four different marine yeasts in the DAPI channel (Figure 4). We found species-specific autofluorescence intensities with autofluorescence clearly visible in *S. dacryoidea*, *R. sphaerocarpa* and *Rhodotorula* sp. In contrast, *M. australis* showed only minor autofluorescence. These results suggest that autofluorescence is not restricted to a single species, but instead might be a widely distributed feature in marine fungi, albeit species-specific. Future research including a larger number of more diverse fungi would be help in confirming whether autofluorescence is a global characteristic of marine fungi.

**Figure 5:**
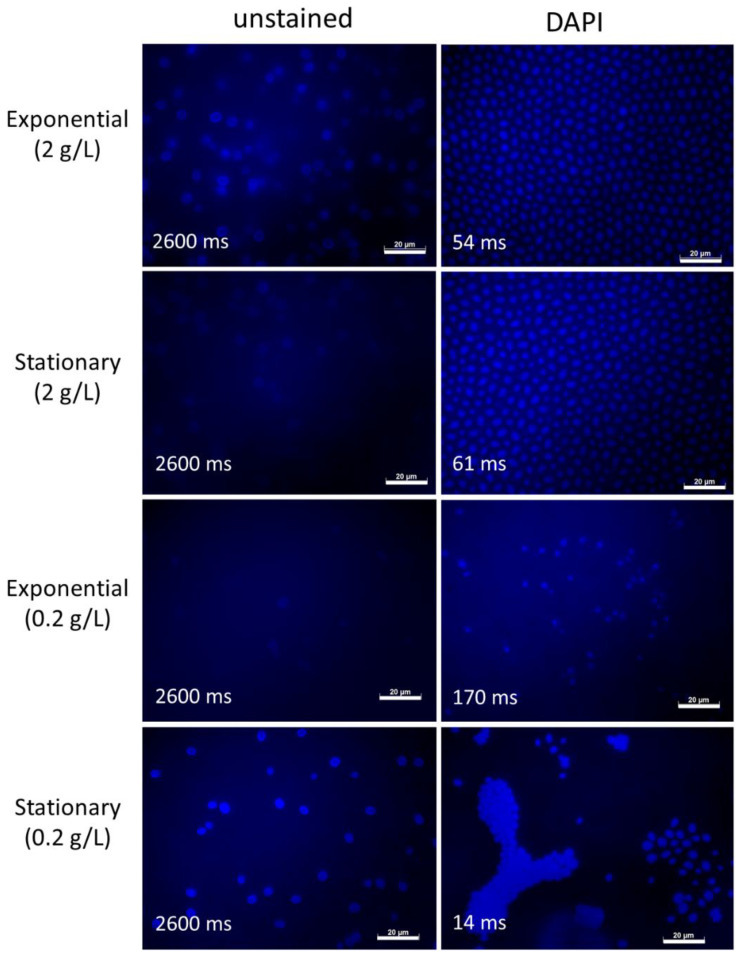
Autofluorescence of *S. dacryoidea* grown in two different nutrient concentrations and sampled in the exponential and stationary phase. Right panels show additional staining with DAPI. Exposure times are depicted in milliseconds (ms). Scale bar = 20 µm.

To study the effect of additional staining with DAPI on the autofluorescence of *S.dacryoidea*, we stained the fungal cells sampled in both growth stages and nutrient conditions with DAPI (Figure 5).

DAPI-staining intensified fungal fluorescence in all conditions (note the shorter exposure time) with strongest fluorescence of the cell nucleus (Figure 5). Hence, although fungal cells are auto-fluorescent, the cell nucleus is still clearly visible and can be distinguished from the rest of the cell when stained with DAPI.

**Figure 6:**
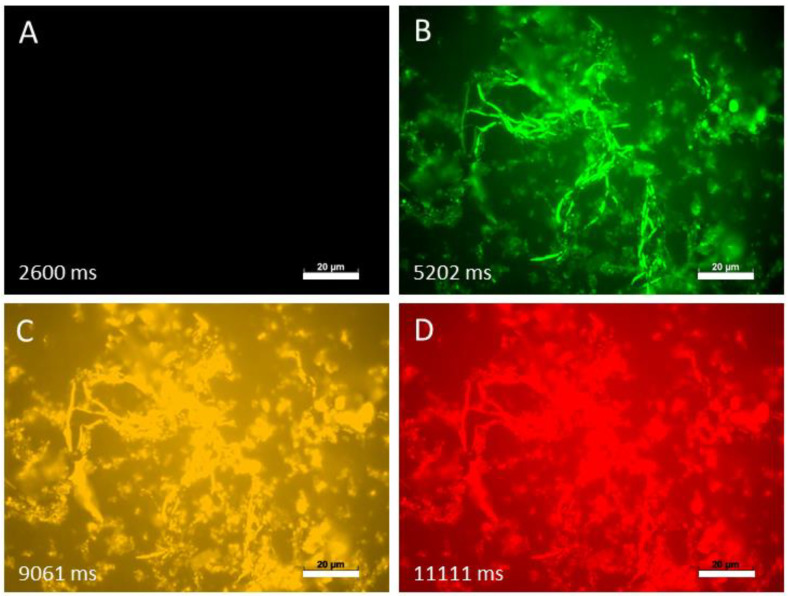
Autofluorescence of *B. parvus*. (**A**): DAPI channel; (**B**): FITC channel; (**C**): DsRed channel; (**D**): rhodamine channel. Picture exposure times are depicted in milliseconds (ms). Scale bar = 20 µm.

Finally, to study fungi with hyphae-morphotype, we investigated the autofluorescence of *Blastobotrys parvus* in four different channels (Figure 6).

When observed with the DAPI channel, *B. parvus* showed no autofluorescence. However, after changing to FITC, DsRed and Rhodamine channel, the autofluorescence of *B. parvus* was clearly visible, supporting that this phenomenon occurs in both fungal morphotypes.

Collectively, this is the first time that autofluorescence is shown in marine fungal species. These results are consistent with previous studies on terrestrial and human-derived fungal species [2,3,4,5,6]. Our results also suggest that the intensity of fungal autofluorescence changes with the excited wavelengths. We also provide a preliminary indication that the autofluorescence of marine fungi varies between fungal species and in relation to the nutrient availability and growth stage. This is consistent with a previous study where the autofluorescence of cells/tissues was suggested to be influenced by the physiological state of the organism [1].

The existence of autofluorescence in marine fungi is important to consider, particularly when dealing with fluorescence-based techniques for analysis or identification. This autofluorescence can be used as a methodological advantage without the need of prior staining as shown for human associated fungi [2,3]. It can be misleading, however, when working with fluorescence microscopy. For instance, a very common method in microbiological studies for identification of cells and estimating their relative abundance is fluorescence in situ hybridisation (FISH). Its principle relies on staining microbial cells with, for example, DAPI, which binds to the DNA in the cells, and also with some specific probe to target a specific taxon. As a result, cell compounds that contain DNA emit a fluorescent signal when exited in a specific wavelength [15,16] and the targeted taxa with the corresponding FISH-probe will also emit fluorescence in a different wavelength to DAPI [17]. Thus, the existence of autofluorescence in marine fungi might lead to potential interferences, potentially resulting in false “positive” results due to natural autofluorescence in marine fungal cells. Therefore, natural autofluorescence in marine fungi should be investigated before applying fluorescence-dependent analytical methods.

## Figures and Tables

**Table 1:** Marine fungi used for the autofluorescence experiments.

Species	Division	Origin of Isolation	ACBR Code
*Metschnikowia australis*	Ascomycota	Antarctic Ocean	HA635
*Rhodotorula sphaerocarpa*	Basidiomycota	Antarctic Ocean, Marguerite Bay	HB738
*Sakaguchia dacryoidea*	Basidiomycota	Antarctic Ocean	HB877
*Blastobotrys parvus*	Ascomycota	Antarctic Ocean	HA1620
*Rhodotorula* sp.	Basidiomycota	Atlantic Ocean	-

**Table 2:** Growth media used for marine yeast liquid cultures.

Chemicals (g/L)	Eutrophic Medium	Oligotrophic Medium
Glucose	2	0.2
Peptone	2	0.2
yeast extract	2	0.2
sea salts	35	35
chloramphenicol	0.5	0.5

**Table 3:** Optical density (OD) of different fungal growth stages.

Growth Stage	OD for Eutrophic Medium	OD for Oligotrophic Medium
Adaptation phase	0.07–0.4	<0.15
Exponential phase	0.4–1.2	0.15–0.27
Stationary phase	>1.2	>0.27

## Data Availability

The raw data supporting the conclusions of this article will be made available by the authors, without undue reservation to any qualified researcher.
